# The management of non-traumatic wrist disorders: A national survey of practice

**DOI:** 10.1177/17589983251372949

**Published:** 2025-08-29

**Authors:** Thomas Mitchell, Nick Hamilton, Sionnadh McLean, Ben Dean, George Peat

**Affiliations:** 1 Centre for Applied Health & Social Care Research, 7314 Sheffield Hallam University, UK; 2 Centre for Sports Engineering Research, 7314 Sheffield Hallam University, UK; 3 Faculty of Health, School of Allied Health Sciences, 2306 Charles Darwin University, Australia; 4 Botnar Research Centre of Musculoskeletal Diseases, University of Oxford, UK

**Keywords:** non-traumatic, wrist injury, wrist pain, survey, clinical practice, conservative management

## Abstract

**Introduction:**

Non-traumatic wrist disorders (NTWD) are commonly encountered across care settings, but current patterns of care and clinicians beliefs about the care they provide remains unclear.

**Objective:**

This study aimed to record management approaches to care for NTWD across clinical groups and care settings.

**Methods:**

Ethical approval was sought for an online cross-sectional survey of clinicians [1 Jul - 1 Nov 2023], comprising 18 questions exploring profession, work setting, exposure to NTWD, alongside diagnostic and management confidence. UK-based musculoskeletal (MSK) clinicians were invited to participate through special interest groups, online forums, social media and professional network emails.

**Results:**

Variability was found in the domains of specificity of diagnosis and confidence in management which relates to exposure and profession. Variability was found in the domains of specificity of diagnosis and confidence in management which relates to exposure, profession and clinical setting. Several Patient Related Objective Measures (PROMS) were used by clinicians to assess treatment effect, set goals, and communicate with patients.

**Conclusion:**

This study provides the first description of UK clinicians management of non-traumatic wrist disorders across professional groups and healthcare settings. As evidence-based management remains elusive, deeper understanding of the clinical decision-making and practice behaviour of clinicians would have value in future studies into NTWD.

## Introduction

Scoping reviews of published and unpublished sources,^1^ patient-facing websites^2^ and an audit of care pathways and guidelines^3^ have revealed a lack of high-quality resource to guide best management for non-traumatic wrist problems. Non-traumatic wrist disorders (NTWD) is a term proposed to refer to these poorly understood conditions which include wrist pain, ganglion, tendinopathies, ulna-sided disorders, osteoarthritis and instabilities,^4^ but exclude the well-researched carpal tunnel syndrome. NTWD are commonly encountered musculoskeletal (MSK) conditions across care settings in the UK,^4–6^ yet the established practice of attributing structural pathology to patient symptoms has been questioned by the presence of non-symptomatic pathology.^7–13^ The best methods of achieving accurate diagnosis for specific non-traumatic wrist problems are unclear as is evidence for the superiority of specific condition management or the best setting for provision of care.^1,13^ Devoting resource to reductive lesion-specific care when its benefits are uncertain has been questioned in other body regions for non-traumatic MSK conditions, with calls for prioritisation of person-centred care to support individuals meet their individual care needs.^14–17^ A key component in achieving this is the adoption of Core Outcome Sets (COS) to allow comparisons of outcomes across different treatments or treatment centres and to facilitates shared decision-making and benchmarking across organisations.^18,19^ This is seen as an opportunity to raise the standards of care and research in the management of people with wrist and hand conditions by Wouters et al. (2021), who created a Core Outcome Set (COS) through their application of the International Consortium for Health Outcomes Measurement.^19^ This survey allows examination of currently used outcome measures.

People experiencing NTWD, as well as those charged with the care, encounter significant obstacles in achieving optimal management. Stakeholders have identified the need for improvements in the domains of diagnosis, management, pathways of care and outcome measures for NTWDs in the United Kingdom which aligned with knowledge gaps from literature mapping and a review of online patient-facing materials.^1,3^ As little is known about how these conditions are currently managed nor how effectively, and as little is known about practice behaviour of clinicians when faced with matching the uncertain evidence-base with their duty of care, further inquiry is arguably necessary.^1,4^ The aim of this survey was to record management approaches for patients with non-traumatic wrist disorders (NTWD) in different UK settings and between different practitioner groups. Specific objectives were:

• To investigate approaches to assessment and treatment of NTWD between practitioner groups.
• To investigate differences in patient related outcome measures (PROMS) usage between practitioner groups and settings.

## Methods

The research proposal was reviewed and approved by Sheffield Hallam University Ethics Committee (Converis reference: ER52704199) on the 1^st^ June 2023.

### Study design

An online cross-sectional survey of clinicians (Online Appendix A) was developed using the Harvard University Program on Survey Research Tip sheet on question wording^20^ and developed to address knowledge gaps identified in a previous literature mapping exercise for NTWD relating the domains of diagnosis, management, pathways of care and outcome measures.^1^ Investigating the domain of ‘pathways of care’ is beyond the scope of this survey and has been addressed in other work.^3^ The survey comprised 18 questions about the participant professional background and work setting, exposure to NTWD within their typical clinical workload, diagnostic and management confidence, the use of PROMS and preferred methods of conservative management (Supplemental Section 1). Questions were comprised of quantitative multiple choice or Likert scales.

### Sampling and recruitment

UK-based practitioners involved in the management of NTWD (including surgeons, therapists, and primary care clinicians) were invited to participate in the survey through a targeted recruitment strategy utilising special interest groups, online forums, social media (LinkedIn and Twitter/X) and the lead author’s professional network through email with a recruitment banner (Online Appendix B). Primary care clinicians were made up from General Practitioners and First Contact Practitioners (FCPs), a UK-unique group of musculoskeletal practitioners specialising in primary care, particularly triage and attempted self-management through education, simple advice and reassurance.^21^ A participant information sheet detailing the purpose and anonymous nature of the survey was displayed on the landing page of the online survey (Online Appendix C). A declaration of informed consent was mandated to allow participants access to the survey instrument.

In setting a target sample, we established received estimates of the total number of hand therapists in secondary care rehabilitation (n = 890), wrist and hand surgeons (n = 700) and FCPs in Primary care (n = 800). We were unable to define the number of generalist musculoskeletal practitioners or General Practitioners (GP’s) who have experience of managing NTWD, negating our ability to perform a power calculation to allow confidence in the generalisability of the survey findings. An expectation of response at 15% of known professional group numbers was considered reasonable based on previous response rates for surveys methods for wrist surgeons, hand therapists and FCP’s which varied between 5.7% and 20.1%.^21–23^ We pooled the total estimated number of professionals in the above groups (2390) giving a target number of responders of 385 at 15% response rate.

### Data collection

The survey instrument was designed and disseminated using Qualtrics^TM^ online survey tool endorsed by the host academic institution, Sheffield Hallam University. Data were collected between 1^st^ July and 1^st^ November 2023.

### Text analysis

Text entries to multi-item questions where participants responded ‘other’ to were analysed by the lead author and the most appropriate answer was chosen where possible or logged as missing data when not. The risk of bias from the lead author’s analysis of text entries was mitigated through discussion with the research team.

### Statistical analysis

Data were analysed according to a pre-specified analysis plan, using descriptive statistics and multivariable regression modelling for available-case analysis and after multiple imputation of missing data. The merging of professional categories and clinical settings into broad groups for analysis was made on a case-by-case basis where narrative responses were recorded (Table 1). Responses were exported from Qualtrics to STATA version 17 (StataCorp, College Station, TX, USA) for all analyses. Anonymised raw data and Stata files used in this publication are accessible for independent analysis on the Open Science Framework: https://osf.io/xks9v/.

**Table 1.** Professional group and primary work setting of survey respondents (n = 330).

	Primary work setting				Total
Professional group	Secondary care	Primary care	Community care	Private practice	
MSK rehabilitation^a^	26	19	48	31	124 (*37%*)
First contact practitioner^b^	1	76	2^e^	1	80 (*24%*)
Surgeon^c^	63	0	1	1	65 *(20%)*
Hand therapist^d^	49	1	7	4	61 *(19%)*
Total	139 (*42%*)	96 (*29%*)	58 *(18%)*	37 (*11%*)	330 (*100%*)

**MSK** = Musculoskeletal.

^a^Includes physiotherapists (n = 116) chiropractors (n = 1), sports therapists (n = 2), and musculoskeletal practitioner (n = 5).

^b^Includes first contact practitioners (n = 77), general practitioners (n = 3), and physician associate (n = 1).

^c^Includes wrist and hand surgeons (n = 40), orthopaedic surgeons (n = 20), plastic surgeon (n = 1), Sports and exercise medicine consultants (n = 3) and general practitioners in secondary care (n = 4).

^d^Includes hand therapists from occupational therapy (n = 31) and physiotherapy (n = 30) professional backgrounds.

^e^Includes community care settings and other MSK service settings (unspecified).

### Imputed data analysis

The proportion of missing data on survey items ranged from 0-26%, with 237 of 330 (72%) eligible respondents providing complete data. Our primary analysis was an available-case analysis. To evaluate the sensitivity of our findings to bias due to selective item non-response, we repeated our analyses after multiple imputation of missing data. Missingness patterns were explored for the assumption of missing at random (MAR) and found to be related in particular to order of questions and to a lesser extent professional group and setting. Using the -mi- commands in Stata, we performed multiple imputation with chained equations (or monotone where appropriate) with 30 imputed datasets,^24^ combined using Rubin’s rules.

## Results

The 330 clinicians who responded were comprised of 124 musculoskeletal rehabilitation clinicians, 80 FCP’s, 65 surgeons and 61 hand therapists). The primary place of work of participants were 139 in secondary care, 96 in primary care, 55 in community care and 37 in private practice.

Our re-analyses on multiply imputed data provided similar findings to the primary available-case analysis (Supplemental Section 2).

The sample population is displayed as merged professional groups and merged work settings in Table 1. Although our target of 385 respondents was not achieved, the sample had broad equality between the known professional group numbers. Most of the surgeon group were based in secondary care and the vast majority of FCP group were in primary care, which is in line with expectations for where delivery of their services would be based.

Table 2 displays mean scores for clinicians estimated contacts for NTWD in the context of their typical workload for each merged professional group. The greater number of patients seen by the First contact practitioner group (53.0 compared to all group 39.4) shorter contact time (20.1 min comparted to all group 24.6 min) and fewer number of follow-up sessions (1.4 sessions compared to all group 2.7 sessions) is in line with expectations of the nature of this role. The proportion of NTWD seen by clinicians varied between the least frequent 7.8% for FCP’s and most frequent at 18.9% for the Surgeon group.

**Table 2.** Professional group estimates for clinical encounters for NTWD.

Professional group	Clinical encounter estimates (Mean scores)				
	NTWD patients seen per fortnight	Total patients seen per week	Proportion of NTWD patients per clinical list	Time spent per contact session (mins)	Number of follow up sessions
MSK rehabilitation	4.9 (5.5) (n = 102)	37.1 (25.6) (n = 100)	13.3% (18.9) (n = 100)	29.2 (9.9) (n = 102)	3.2 (1.6) (n = 102)
First contact Practitioner	5.8 (4.0) (n = 72)	53.0 (27.8) (n = 69)	7.8% (8.9) (n = 69)	20.1 (6.3) (n = 72)	1.4 (1.2) (n = 72)
Surgeon	10.7 (8.9) (n = 58)	35.1 (20.1) (n = 57)	18.9% (16.0) (n = 57)	15.3 (5.6) (n = 58)	2.1 (1.0) (n = 58)
Hand therapist	6.3 (3.9) (n = 55)	30.8 (19.2) (n = 55)	17.1% (18.6) (n = 55)	30.6 (7.3) (n = 55)	4.2 (1.3) (n = 55)
All professional groups	6.6 (6.2) (n = 287)	39.4 (25.3) (n = 281)	13.8% (8.9) (n = 281)	24.6 (9.8) (n = 287)	2.7 (1.6) (n = 287)

Figures are mean (SD)

**Figure 1.**
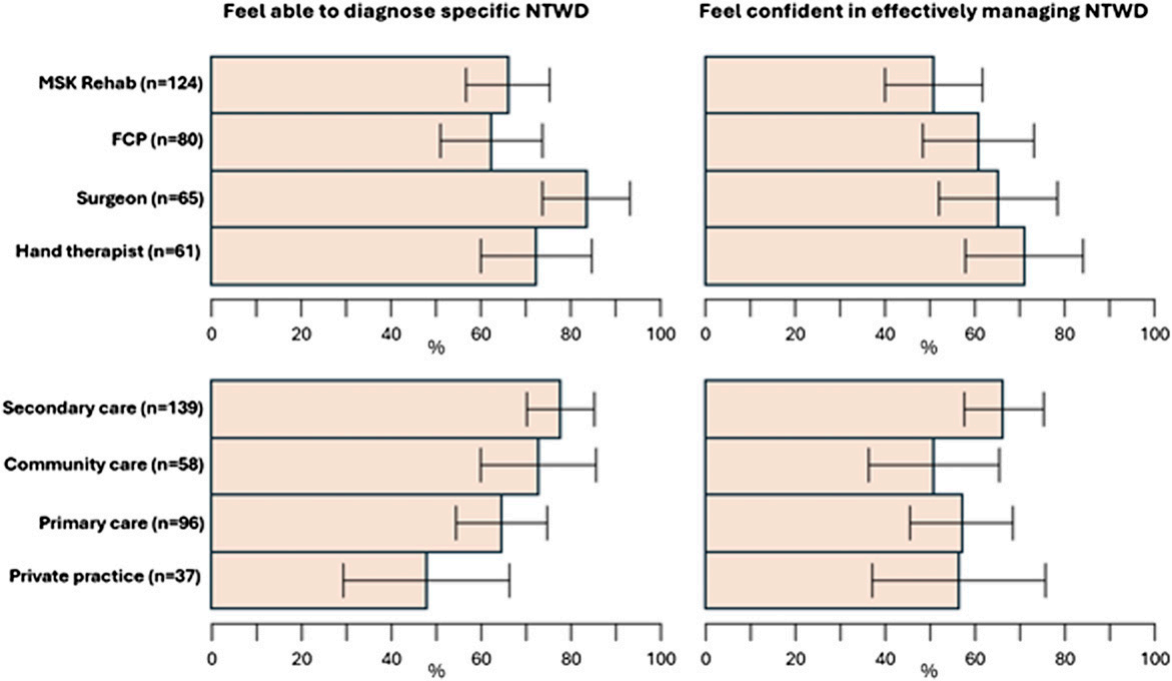
Bar chart displaying clinician (upper row) and clinical setting (lower row) data for ability to reach specific diagnosis, confidence in management.

Variation in perceived diagnostic ability and confidence in providing effective management were found (Figure 1). 70% of respondents felt able to diagnose specific NTWDs; 59% felt ‘completely’ or ‘fairly’ confident in providing effective management. The most confident professional group in reaching specific diagnosis was surgeons (84%), whilst least confident were FCPs (63%). Across settings, private practice and primary care clinicians felt less able to reach a specific diagnosis, though the span of confidence intervals is broad within groups and between settings. The professional group who had least confidence in their ability to manage NTWD were MSK rehabilitation (52%) while 71% of hand therapists and 64% of hand surgeons were ‘Completely/ Fairly confident’. Multivariable logistic regression analysis identified that NTWD caseload was positively associated with perceived diagnostic ability and with management confidence (adjusted odds ratio 1.13; 95%CI: 1.06, 1.22 and 1.09; 1.03, 1.16 respectively).

Clinical diagnostic methods rated as most useful were subjective questioning, symptom reproduction and palpation, while few clinicians found usefulness in bodychart/heatmap methods. MRI, X-ray and Ultrasound scan were rated the most useful advanced diagnostic means for NTWD, with nerve conduction studies, CT scan and diagnostic arthroscopy less favoured.

Amongst those clinicians who felt able to make specific diagnosis of NTWD, diagnostic confidence was highest for de Quervain’s and lowest for wrist instability and ulna-sided wrist problems. Local exercise prescription and self-management were the most recommended amongst a broad range of treatments.

Use of outcome measures was uncommon among FCPs and surgeons and in the primary care setting. Hand therapists were the most frequent users, as were those in secondary care and private practice settings.

Variability was found in the types and usage of PROMS. Among those using outcomes, grip strength and simple pain VAS/NRS were most common, and typically used to assess treatment effect, set goals, and communicate with patients.

## Discussion

**Figure 2.**
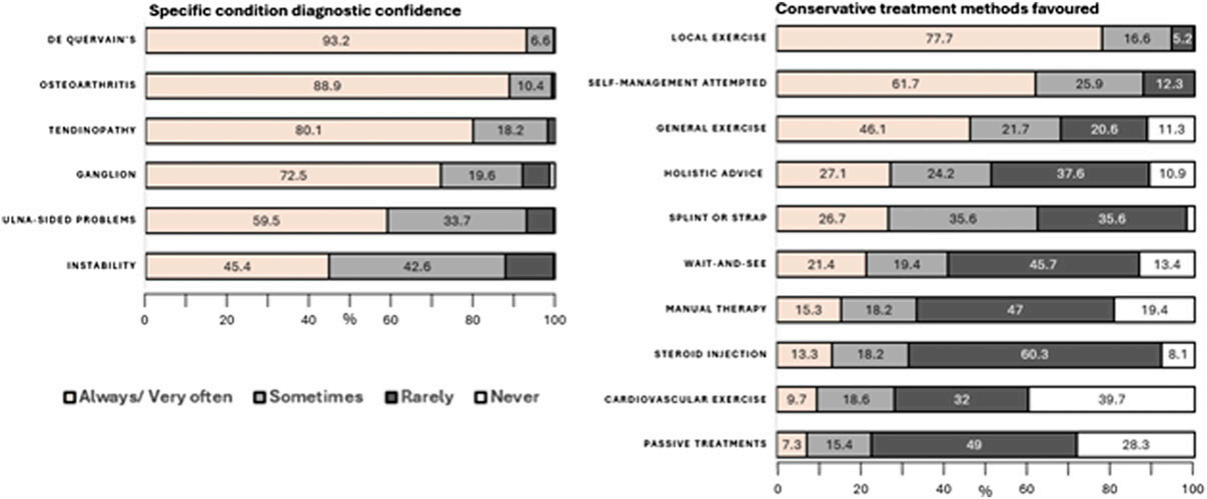
Stacked bar chart displaying clinician confidence in reaching diagnosis for specific NTWD conditions and favoured conservative treatments.

**Figure 3.**
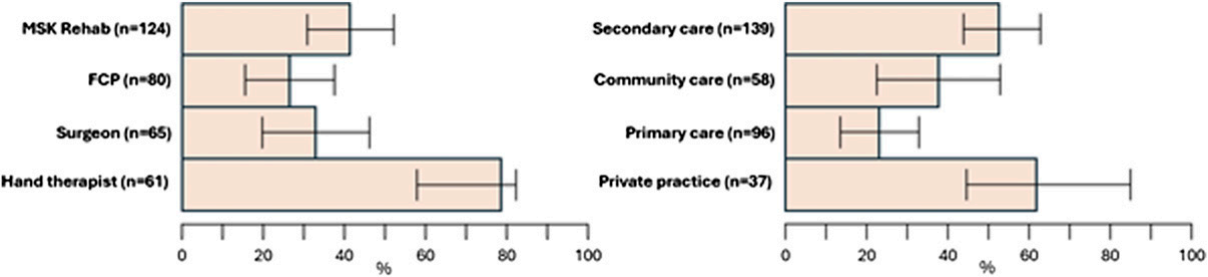
Bar chart displaying use of outcome measures by clinical grouping and setting.

This survey is the first to record current management of people with NTWD in the UK, providing a resource to which care providers can compare their services. Further investigation revealed that while the majority of respondents felt confident in making specific NTWD diagnoses, this varied by professional group, by main care setting, and by specific NTWD (Figure 1). Our findings suggest that diagnostic confidence is likely to be lowest among FCPs, those working in primary care or private practice, and for wrist instability and ulna-sided wrist problems. A large variety of treatment options (Figure 2) and PROMS (Figure 3, Table 3) are being used in the management of NTWD. Our findings were largely concordant with recent reviews of the published and unpublished evidence-base and audit of care pathways for NTWD.^1–3^

**Table 3.** Usage of specific outcome measures and their justification.

Outcome measures used	n	%
VAS (n = 256)	55	21.5
Grip strength (n = 256)	72	28.1
NRS (n = 256)	49	19.1
PSFS (n = 256)	42	16.3
QuickDash/Dash (n = 256)	46	18.0
PRWE (n = 256)	32	12.5
Euroqual (EQ5d) (n = 256)	10	3.9
SF36/12 (n = 256)	3	1.2
Orebro (n = 256)	1	0.4

Reasons for using outcome measures	n	%
Assessment of treatment (n = 256)	90	35.1
Setting treatment goals (n = 256)	66	25.8
Communicating with patients (n = 256)	65	25.4
Audit of service delivery (n = 256)	46	18.0
Fulfilling standards (n = 256)	41	16.0
Communicating with other clinicians (n = 256)	41	16.0
Prognosis (n = 256)	41	16.0
Research activities (n = 256)	22	8.6
Commissioner’s requirements (n = 256)	13	5.1
Medicolegal requirements (n = 256)	16	6.2
Marketing (n = 256)	1	0.4

**Figure 4.**
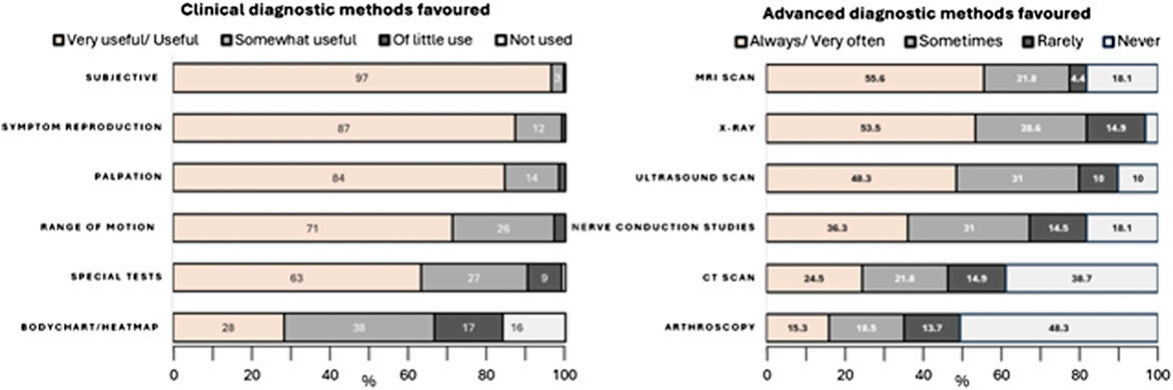
Stacked bar chart displaying favoured methods of clinical and advanced diagnostic methods.

Expanding on our first objective to investigate approaches to care across practitioner groups and settings, our regression analysis found perceived diagnostic ability and management confidence were positively associated with NTWD caseload (Figure 1). The reasons for the correlation between clinicians’ confidence in managing NTWD cases and the number of such cases they encounter is unknown. One possibility is that this reflects a general increase in their expertise in handling all wrist problems. Alternatively, it could signify improvements in their specific diagnostic and management skills for NTWD with more experience. A significant minority of clinicians do not feel able to navigate toward specific diagnosis for NTWD at all (Figure 1), and clinical assessment techniques across groups appear to favour subjective, non-specific and functional measures rather than specific lesion-based tests (Figure 4), indicating a pragmatic approach is used to navigating toward diagnosis. Whether specific diagnosis is needed for the effective management of NTWD (once serious pathology or orthopaedic referral has been ruled out) is a matter of debate, and non-specific management based on person-centred care has been recommended for non-traumatic disorders in other MSK areas including low back, shoulder and knee.^14–16^ Interestingly the least confident group in achieving reductive diagnosis were FCP’s, however they were found to be more confident in managing NTWD than MSK Rehab practitioners, implying that the lack of specific diagnosis may not impact upon clinicians confidence in effective management. This might reflect the place of primary care in the pyramid of health systems where subjective observation, basic examination, reassurance, simple advice and knowing when to escalate are core competencies,^25^ which may differ to settings where reductive examination and methods of advanced diagnostics and specialist management are provided. Currently there is no evidence to suggest the superiority of clinical setting or professional specialisation in the management of NTWD,^1^ an area which would benefit from further examination to inform future care resource allocation.

Of those who felt they could be specific to lesion-based diagnosis, the variability in perceived diagnostic accuracy between conditions reflects limitations of the means of assessment for instability and ulna sided pain found in the literature,^11–13,26^ in particular the uncertain accuracy of special tests, and the ordering and weighting needed to achieve clinical diagnosis. Whether the confidently diagnosed de Quervain’s should be removed from the proposed grouped NTWD category requires further thought, especially in cases which exhibit acute and straightforward features. The diagnostic confidence of clinicians does not equate to high accuracy of diagnostic means particularly when the commonly utilised Finkelstein´s and Eichhoff´s special tests revealed false positives, of 46,7% and 53,3% respectively in one review,^27^ raising questions of whether the success of specific management related to these diagnoses can be attributed to intended effects. In cases with concordant presentations with symptom overlap and incalcitrant presentations^4,28^ where specific condition management has failed to achieve acceptable outcomes, it is reasonable to prioritise a non-specific approach based on the principles of person-centred care.

The array and application of PROMS found in achieving our second objective indicates more scrutiny into their application is indicated (Table 3). The regular COS for wrist conditions is comprised of PSFS, NPRS EQ5d and the PRWHE, with the extended COS including grip strength.^19^ While some of these PROMS were used, others were also applied, and it is unclear how comprehensively the COS was followed. Addressing the lack of guidelines for management of NTWD^3^ and encouraging the use of the wrist COS in their composition would appear to hold benefits in gravitating toward high-value care . In examining differences in the use of PROM between practitioner groups and settings, it is unclear whether the use of outcome measures are currently important in the journey of people with NTWD as most people with these conditions are likely to be managed in primary care where they are seldom utilised, and the motivation for their use in secondary care often includes non-clinical data gathering (Table 3).

MSK conditions form an increasing burden of resource for healthcare providers,^15,16^ and our survey found NTWD formed proportions of clinical contact time across groups in line with expectations of the care burden,,^5,6^ reflecting demand for effective management.^1^ Non-traumatic musculoskeletal conditions challenge the traditional lesion-specific approach to management in ways that traumatic conditions often do not, across all body regions. There is a compelling case for shifting the focus from diagnosis-driven treatment to a broader, more holistic approach that prioritises the individual’s overall well-being and unique needs. This perspective aligns closely with the principles of person-centred care. The adoption of COS for the wrist holds promise in allowing benefits for both research and development of high-value care as measures would allow tracking of improvements by function over structural considerations.

### Strengths and limitations

In the absence of a fully enumerated sampling frame of UK MSK clinicians, it is difficult to judge potential response and selection bias. However, the number of survey respondents approached the target sample and compares favourably to similar publications of the professional groups studied in this work.^21–23^ The characteristics of the sample matched expected features of setting, consultation duration and number of appointments indicating a level of confidence can be made in the authenticity of data reflecting professional groupings and clinical settings. Other strengths include data analysis being conducted in accordance with a pre-specified analysis plan, and consistency of findings following the use of multiple imputation. Anonymous, record-level data from the survey is freely available on the Open Science Framework. A limitation was the lack of a power calculation, and more data may have allowed regression analysis within different practitioner groups and settings, and how these affected their preferences for assessment and management techniques. Our cross-sectional survey did not seek to explore the relationship between clinician confidence, care provided, and patient outcomes. This could be the focus of a future study although the variability and absence of patient-reported outcome measurement found in this study would limit ‘real world’ evaluation in routine practice in the UK.

## Conclusion

In this survey of healthcare practitioners who treat NTWD, it was found that variability between clinical groupings and work setting exists, notably in diagnostic confidence and confidence in management. The usage of outcome measures varied by care setting and in types selected. Deeper understanding of the clinical decision-making and practice behaviour of clinicians considering the poor evidence-base would have value in future studies into NTWD.

## Supplemental Material

Supplemental Material - The management of non-traumatic wrist disorders: A national survey of practice Supplemental Material for The management of non-traumatic wrist disorders: A national survey of practice by Thomas Mitchell, Nick Hamilton, Sionnadh McLean, Ben Dean, George Peat in Hand Therapy.

Supplemental Material - The management of non-traumatic wrist disorders: A national survey of practice Supplemental Material for The management of non-traumatic wrist disorders: A national survey of practice by Thomas Mitchell, Nick Hamilton, Sionnadh McLean, Ben Dean, George Peat in Hand Therapy.
