# Supplementary material for: The management of non-traumatic wrist disorders: A national survey of practice
Source: Hand Ther. 2025 Aug 29;31(1):54–61. doi: 10.1177/17589983251372949 (PMC12397095; doi:10.1177/17589983251372949)
Supplement: Supplemental Material - The management of non-traumatic wrist disorders: A national survey of practice [file sj-pdf-1-hth-10.1177_17589983251372949.pdf]

SUPPLEMENTARY DATA

Mitchell T, Hamilton N, McLean S, Dean B, Peat G. The management of non-traumatic wrist disorders: A national survey of practice.

Sensitivity analyses on multiply imputed data

Table S1. Missing data, by survey item

Item	N(%)		Item	N(%)		Item	N(%)
NTWDpts2w	43 (13%)		PROMNRS	74 (22%)		UsefulInve~y	82 (25%)
Allptwk	49 (15%)		PROMPSFS	74 (22%)		UsefulInv~US	82 (25%)
Timespent	43 (13%)		PROMQDASH	74 (22%)		UsefulInve~I	82 (25%)
Followups	43 (13%)		PROMPRWE	74 (22%)		UsefulInve~T	82 (25%)
SpecdiagnN~D	46 (14%)		PROMGRIP	74 (22%)		UsefulInv~CS	82 (25%)
DiagnConfG~a	60 (18%)		PROMEQ5D	74 (22%)		UsefulInve~c	82 (25%)
DiagnConfT~n	61 (18%)		PROMOREBRO	74 (22%)		RecTreatLo~x	83 (25%)
DiagnConfDeQ	61 (18%)		PROMSF	74 (22%)		RecTreatGe~x	83 (25%)
DiagnConfI~b	60 (18%)		PROMUseGoa~t	74 (22%)		RecTreatMa~r	83 (25%)
DiagnConfU~j	61 (18%)		PROMUseCom~t	74 (22%)		RecTreatSt~j	83 (25%)
DiagnConfOA	61 (18%)		PROMUseSta~s	74 (22%)		RecTreatPa~e	83 (25%)
Usefulass~ct	72 (22%)		PROMUseCom~n	74 (22%)		RecTreatWa~e	83 (25%)
Usefulass~at	72 (22%)		PROMUseMed~g	74 (22%)		RecTreatSp~t	83 (25%)
Usefulasse~M	72 (22%)		PROMUseRes~h	74 (22%)		RecTreatHo~c	83 (25%)
Usefulasse~n	72 (22%)		PROMUseMar~t	74 (22%)		RecTreatCVEx	83 (25%)
Usefulass~lt	72 (22%)		PROMUseAudit	74 (22%)		EscalateNTWD	87 (26%)
Usefulasse~m	72 (22%)		PROMUseAss~t	74 (22%)		SelfManagN~D	87 (26%)
UseOutcomes	72 (22%)		PROMUseProg	74 (22%)		ManagConfN~D	87 (26%)
PROMVAS	74 (22%)		PROMUseCom~s	74 (22%)			

Table S2. Complete cases, by professional group and primary work setting

		Complete case
	N	n (%)
Total	330	237 (71.8)
Professional group:		
MSK rehabilitation	124	79 (63.7)
First contact practitioner	80	62 (77.5)
Surgeon	65	48 (73.9)
Hand therapist	61	48 (78.7)
Clinical setting:		
Secondary care	139	100 (71.9)
Community care	58	35 (60.4)
Primary care	96	76 (79.2)
Private practice	37	26 (70.3)

Table S3. Professional group estimates for clinical encounters for NTWD (multiply imputed data)

		NTWD patients seen per fortnight	Total patients seen per week	Proportion of NTWD patients per clinical list	Time spent per contact session (mins)	Number of follow up sessions
	N	Mean (SD)	Mean (SD)		Mean (SD)	Mean (SD)
All professional groups	330	6.5 (6.0)	39.2 (24.3)	Not imputed	24.8 (10.4)	2.8 (1.9)
MSK rehabilitation	124	4.9 (5.2)	36.9 (26.2)	Not imputed	29.1 (11.3)	3.2 (1.8)
First contact practitioner	80	5.9 (4.0)	53.7 (27.0)	Not imputed	20.5 (7.1)	1.4 (1.3)
Surgeon	65	10.5 (8.8)	34.7 (19.8)	Not imputed	16.0 (9.4)	2.2 (5.1)
Hand therapist	61	6.3 (4.0)	30.9 (18.6)	Not imputed	30.3 (8.0)	4.2 (1.6)

Table S4. Professional groups belief in their ability to make specific diagnosis for NTWD (multiply imputed data)

		Able to make specific diagnoses for NTWD	
	N	%	95%CI
All professional groups	330	69.7	(64.3, 75.1)
MSK rehabilitation	124	66.0	(56.7, 75.3)
First contact practitioner	80	62.3	(51.0, 73.7)
Surgeon	65	83.5	(73.7, 93.2)
Hand therapist	61	72.3	(60.0, 84.7)
Clinical setting:			
Secondary care	139	77.8	(70.3, 85.3)
Community care	58	72.8	(60.0, 85.7)
Primary care	96	64.7	(54.5, 74.8)
Private practice	37	47.9	(29.4, 66.4)

Table S5. Association between belief in ability to diagnose specific NTWD and professional group, clinical setting, and number of NTWD patients seen (multiply imputed data, n=330)

	aOR	(95%CI)
Professional group:		
Surgeon	1	
Hand therapist	0.70	(0.27, 1.85)
MSK rehabilitation	0.65	(0.22, 1.95)
First contact practitioner	0.36	(0.09, 1.54)
Clinical setting:		
Secondary care	1	
Community care	1.29	(0.47, 3.52)
Primary care	1.23	(0.36, 4.17)
Private practice	0.41	(0.15, 1.14)
Number of NTWD patients seen in past 2 weeks	1.11	(1.03, 1.20)
aOR Adjusted odds ratio		

Table S6. Clinician confidence in reaching specific diagnosis for various NTWD among professionals who believed in their ability to make specific NTWD diagnoses (multiply imputed data, n=330)

	Always	Very often	Sometimes	Rarely	Never
NTWD condition:					
Ganglion	19.2	53.3	19.6	6.7	1.2
Tendinopathy	17.2	62.9	18.2	1.8	0
De Quervain's	39.4	53.8	6.6	0	0.2
Instability	4.4	41.0	42.6	11.8	0.1
Ulna-sided wrist problems	9.1	50.4	33.7	6.6	0.2
Osteoarthritis	20.1	68.8	10.4	0.7	0

Table S7. Usefulness ratings of various clinical diagnostic methods (multiply imputed data, n=330)

	Very useful	Useful	Somewhat useful	Of little use	Not used
Clinical diagnostic method:					
Subjective	80.5	15.9	3.1	0.5	0
Palpation	40.0	44.5	14.0	1.6	0
Range of motion	24.4	46.6	25.9	3.2	0
Bodychart/heatmap	7.7	21.3	38.2	17.2	15.6
Special tests	21.9	41.0	27.3	9.0	0.8
Symptom reproduction	47.8	39.0	12.3	0.9	0

Table S8. Usefulness ratings of advanced diagnostic methods (multiply imputed data, n=330)

	Always	Very often	Sometimes	Rarely	Never
Advanced diagnostic method:					
X-ray	25.3	27.7	28.6	15.1	3.3
Ultrasound scan	19.3	30.0	30.7	9.8	10.2
MRI scan	31.3	24.5	21.5	4.4	18.3
CT scan	11.8	13.5	21.5	14.4	38.8
Nerve conduction studies	14.2	21.8	31.0	14.4	18.5
Arthroscopy	7.7	8.8	18.3	13.7	51.4

Table S9. Level of confidence of clinicians in managing NTWD (multiply imputed data, n=330)

	N	Not at all confident	Slightly confident	Somewhat confident	Fairly confident	Completely confident
All professional groups	330	1.3	12.1	26.5	51.1	9.0
MSK rehabilitation	124	2.3	17.2	29.4`	47.0	4.2
First contact practitioner	80	1.7	12.4	24.4	58.0	3.5
Surgeon	65	0	2.4	32.8	42.3	22.6
Hand therapist	61	0	11.8	16.8	59.8	11.5

Table S9a. 'Completely' or 'fairly' confident in managing NTWD (multiply imputed data, n=330)

		'Completely' or 'fairly' confident in effectively managing NTWD	
	N	%	95%CI
All professional groups	330	60.0	(53.8, 65.8)
MSK rehabilitation	124	50.9	(40.0, 61.7)
First contact practitioner	80	60.8	(48.4, 73.2)
Surgeon	65	65.2	(52.0, 78.4)
Hand therapist	61	71.0	(57.9, 84.1)
Clinical setting:			
Secondary care	139	66.2	(57.4, 75.1)
Community care	58	50.9	(36.4, 65.5)
Primary care	96	57.2	(45.6, 68.7)
Private practice	37	56.5	(37.2, 75.8)

Table S10. Recommended conservative treatment for NTWD (multiply imputed data, n=330)

	Always	Very often	Sometimes	Rarely	Never
Recommended conservative treatment:					
Local exercise	38.9	39.2	16.0	5.3	5.3
General exercise	20.5	26.0	22.0	20.1	11.5
Manual therapy	4.1	11.8	18.7	46.1	19.3
Steroid injection	1.4	11.9	18.0	60.0	8.7
Passive treatments (wax baths, dry needling, electrotherapy, heat, ice)	1.7	6.5	15.3	47.7	28.8
Wait-and-see	1.0	20.5	18.9	45.9	13.7
Splint or strap	2.9	23.1	35.6	36.4	2.1
Holistic advice (weight loss, healthy eating, smoking cessation etc)	12.4	15.0	24.0	37.6	11.0
Cardiovascular exercise	2.4	7.5	18.8	32.0	39.3
Self-management attempted	15.3	46.1	25.7	12.9	0

Table S11. Use of outcome measures, overall and by professional group and primary work setting (multiply imputed data)

Use Outcome Measures	N	%	95%CI
All	330	43.0	(36.7, 49.3)
Professional group:			
MSK rehabilitation	124	41.4	(30.9, 51.2)
First contact practitioner	80	26.6	(15.6, 37.6)
Surgeon	65	33.0	(19.8, 46.2)
Hand therapist	61	78.6	(66.4, 90.8)
Clinical setting:			
Secondary care	139	52.6	(43.1, 62.0)
Community care	58	37.8	(22.6, 53.0)
Primary care	96	23.3	(13.6, 33.0)
Private practice	37	61.8	(42.1, 81.5)

Table S12. Use of outcome measures and justification of use (multiply imputed data, n=330)

	%	(95%CI)
Outcome measures used:		
Grip strength	29.0	(23.4, 34.6)
VAS	22.7	(17.2, 28.3)
NRS	20.0	(15.2, 24.8)
QuickDash/Dash	18.7	(13.5, 23.9)
PSFS	17.0	(12.2, 21.8)
PRWE	13.3	(9.2, 17.4)
EuroQual (EQ5D)	4.2	(1.6, 6.9)
SF36/12	1.9	(0, 3.9)
Orebro	1.1	(0, 2.9)
Reasons for using outcome measures:		
Assessment of treatment	36.7	(30.7, 42.8)
Setting treatment goals	27.0	(21.6, 32.5)
Communicating with patients	26.3	(20.9, 31.7)
Audit of service delivery	19.0	(14.0, 24.1)
Prognosis	17.2	(12.5, 21.9)
Fulfilling standards	16.5	(11.8, 21.2)
Communicating with other clinicians	16.5	(11.8, 21.3)
Research activities	9.2	(5.2, 13.1)
Medicolegal requirements	6.9	(3.4, 10.3)
Commissioner’s requirements	6.6	(2.6, 10.7)
Marketing	1.3	(0, 3.3)

EQ5D EuroQual 5-dimension qualify life; **NRS** Numerical Rating Scale; **PRWE** Patient Rated Wrist Evaluation; **PSFS** Patient Specific Functional Scale; **VAS** Visual Analogue Scale
