# Supplementary material for: The management of non-traumatic wrist disorders: A national survey of practice
Source: Hand Ther. 2025 Aug 29;31(1):54–61. doi: 10.1177/17589983251372949 (PMC12397095; doi:10.1177/17589983251372949)
Supplement: Supplemental Material - The management of non-traumatic wrist disorders: A national survey of practice [file sj-pdf-2-hth-10.1177_17589983251372949.pdf]

This survey will help us to understand how patients with non-traumatic wrist disorders are managed in different health settings and by different practitioner types. It should take 5-10 minutes to complete, and your participation will be anonymous.

Non-Traumatic Wrist Disorders (NTWD) are made up of various conditions including non-specific pain, instability, osteoarthritis, ganglia, ulna-sided pain, tendon problems and other disorders. This survey will ask about your management of these. Wrist conditions not included in this study are carpal tunnel syndrome, traumatic wrist injuries or inflammatory presentations and hand problems.

The completion of this questionnaire is voluntary and all the information that you give us will be treated with the strictest confidence.

For any concerns or queries, please contact survey lead Thomas Mitchell by phone on +4407976931983 or email tm8999@hallam.shu.ac.uk, or the lead supervisor Dr. Sionnadh McLean by email s.mclean@shu.ac.uk.

Occupational questions

What is your professional grouping?

- ◯ Wrist and Hand Surgeon
- ◯ First Contact Practitioner
- ◯ General practitioner
- ◯ Hand therapist (OT)
- ◯ Hand therapist (PT)
- ◯ Orthopaedic surgeon
- ◯ Physiotherapist
- ◯ Plastic surgeon

Other, please specify

What is your primary work setting?

- ◯ Secondary care/hospital
- ◯ Community musculoskeletal service
- ◯ Primary care centre/GP practice
- ◯ Private practice
- ◯ Interface musculoskeletal service

Other, please specify

Current practice questions

How many NTWD cases have you seen in the last two-weeks?

	0	2	4	6	8	10	12	14	16	18	20
Patients seen with NTWD											

On average, how long do you spend with a patient?

	0	5	10	15	20	25	30	35	40	45	50	55	60
Minutes													

Can you estimate how many times would you follow up your patients?

	1	2	3	4	5	6	6	7	8	9	10
Number of contacts											

How many patients are on your clinical list in a standard week?

	0	10	20	30	40	50	60	70	80	90	100
Number of patients/week											

For each of the following clinical assessments methods, which do you find useful for patients with NTWD?

	Very useful	Useful	Somewhat useful	Of little use	Not used
Subjective questioning/history	◯	◯	◯	◯	◯
Self-reported pain	◯	◯	◯	◯	◯
Palpation	◯	◯	◯	◯	◯
Manual accessory motion	◯	◯	◯	◯	◯
Range of motion	◯	◯	◯	◯	◯
Visualisation of pain using heatmaps	◯	◯	◯	◯	◯
Functional tests	◯	◯	◯	◯	◯
Special tests	◯	◯	◯	◯	◯

If you use other clinical methods of diagnosing NTWD, please give details below:

Do you use Patient Related Outcome Measures (PROMS) for NTWD?

◯ Yes

◯ No

If yes, please specify which you use:

For each of the following investigations do you find useful in confirming a diagnosis of NTWD?

	Very useful	Useful	Somewhat useful	Of little use	Not used
X-ray/radiograph	◯	◯	◯	◯	◯
Ultrasound scan	◯	◯	◯	◯	◯
MRI/MRA scan	◯	◯	◯	◯	◯
CT scan	◯	◯	◯	◯	◯
Nerve conduction study	◯	◯	◯	◯	◯
Arthroscopy	◯	◯	◯	◯	◯

If you use other diagnostic methods, please record them in the box below:

Which conservative interventions would you recommend for first-line and second-line management of NTWD?

	First line management	Second line management
Injection	☐	☐
Splint	☐	☐
Local exercise	☐	☐
Activity modification	☐	☐
Manual therapy	☐	☐
Global exercise	☐	☐
Sensorimotor training	☐	☐
Proprioceptive training	☐	☐
Heat	☐	☐
Ice	☐	☐
Ultrasound	☐	☐
Taping	☐	☐
Acupuncture	☐	☐
Wax bath	☐	☐
Lazer	☐	☐
Watchful wait	☐	☐
TENS	☐	☐

If you recommend other conservative treatment methods, please record them in the box below:

Please think about your clinical decision-making and click on the the box which fits your position best:

	Always	Very often	Often	Sometimes	Never
How often do you escalate patients with NTWD to diagnostics, onward referral, or invasive management?	○	○	○	○	○
How often do you think you attempt self-management for patients with NTWD?	○	○	○	○	○

What level of confidence do you have in managing patients with NTWD?

- ◯ Not confident at all
- ◯ Slightly confident
- ◯ Somewhat confident
- ◯ Fairly condfident
- ◯ Completely confident

Block 3

If you would be interested in being interviewed about your opinions and experiences in managing patients with non-traumatic wrist disorders, please leave your email address below.

Email address

Thank you for taking this survey.

If you know any other UK surgeons, therapists or primary care clinicians with who see patients with non-traumatic wrist disorders, we would be grateful if you could forward on the link to this survey:

Link: www.qualtrics.com/survey_ehV1KgIlk3NHSrfW

Please click the "Next" button to submit and record your responses.
